# Atmospheric aerosol growth rates at different background station types

**DOI:** 10.1007/s11356-020-11424-5

**Published:** 2020-11-12

**Authors:** Adéla Holubová Šmejkalová, Naděžda Zíková, Vladimír Ždímal, Helena Plachá, Miroslav Bitter

**Affiliations:** 1 Czech Hydrometeorological Institute, Na Šabatce 2050/17, 143 06 Prague 4-Komořany, Czech Republic; 2 Air Quality Department, Košetice Observatory, Czech Hydrometeorological Institute, 39422 Košetice, Czech Republic; 3 Institute for Environmental Studies, Faculty of Science, Charles University, Benátská 2, 128 01 Prague 2, Czech Republic; 4 Institute of Chemical Process Fundamentals, CAS, Rozvojová 135, 165 02 Prague 6, Czech Republic

**Keywords:** Growth rate, Condensation sink, New particle formation, Source location estimation, Pollution load, Background station

## Abstract

Highly time-resolved particle number size distributions (PNSDs) were evaluated during 5 years (2013–2017) at four background stations in the Czech Republic located in different types of environments—urban background (Ústí nad Labem), industrial background (Lom), agricultural background (National Atmospheric Observatory Košetice), and suburban background (Prague-Suchdol). The PNSD data was used for new particle formation event determination as well as growth rate (GR) and condensation sink (CS) calculations. The differences or similarities of these parameters were evaluated from perspectives of the different pollution load, meteorological condition, and regional or long-range transport. The median growth rate (4 nm h^−1^) is very similar at all stations, and the most frequent length of growth lasted between 2 and 4 h. Condensation sink reflects the pollution load at the individual station and their connection to the environment type. The highest median, CS = 1.34 × 10^−2^ s^−1^, was recorded at the urban station (Ústí nad Labem), and the lowest (CS = 0.85 × 10^−2^ s^−1^) was recorded at the agricultural station (National Atmospheric Observatory Košetice). Conditional probability function polar plots illustrate the influence of source location to GR. These primary potential emission sources involve traffic, operation of a power plant, and domestic heating.

**Supplementary Information:**

The online version contains supplementary material available at 10.1007/s11356-020-11424-5.

## Introduction

Atmospheric aerosols are ubiquitous particles, and their presence in the atmosphere contributes to climate change patterns (Kulmala et al. 2004a). Aerosols affect the climate through direct and indirect effects. Atmospheric aerosols can directly scatter and/or absorb solar radiation and thus directly affect the Earth’s radiation balance. The radiation budget is also affected indirectly by aerosols altering clouds’ optical properties and their lifetime, with aerosol acting as cloud condensation nuclei (CCN) (Kulmala et al. 2004b; Pöschl 2005; Yli-Juuti et al. 2011; Stocker et al. 2013).

Nonetheless, the role of aerosols in the climatic system still includes uncertainties strongly influencing model simulations (Zhao et al. 2018). One of the uncertainties is caused by the secondary aerosol formation and its consequent growth, called new particle formation (NPF) event. The process of NPF is favored by the presence of sulfuric acid, and low-volatile oxidized organic vapors; on the contrary, the NPF suppressing factor can be a high amount of pre-existing particles in the atmosphere (Dada et al. 2017; Ling et al. 2019). The favoring or suppressing NPF conditions are very closely connected with the behavior of the particle growth rate (GR) and condensation sink (CS).

The GR reflects the sum of all gas-to-particle conversion processes. It contains essential information on the chemical processes that affect growth (Kuang et al. 2012).

The CS values are influenced by the atmospheric pollution load of existing particles and gas precursors. Large CS is a result of the scavenging of freshly nucleated particles and condensable vapors by existing particles (Zhang et al. 2016). It is still not clear if the high amount of pre-existing particles has a positive or negative effect on NPF, however (Hamed et al. 2007; Nie et al. 2014; Zhang et al. 2016; Zhao et al. 2018; Chu et al. 2019). Since the level of condensation sink is generally higher in more polluted areas as compared to a clean environment (Kulmala et al. 2004a; Pushpawela et al. 2018; Chu et al. 2019), the CS could be marked as an indicator for NPF occurrence (Pikridas et al. 2015; Zhang et al. 2016). Polluted locations, however, contain an abundance of condensable materials resulting in the possibility of observing increased CS along with an elevated number of NPF in these environments (Kulmala et al. 2017; Bousiotis et al. 2019).

The dependency of GR on particle size was recorded (Pushpawela et al. 2018) as well as the relation to pollution loads (Jeong et al. 2010; Bousiotis et al. 2019). Nevertheless, anthropogenic pollution is not the only influencer for GR values. Apart from pollution in cities, other condensation materials supporting GR like volatile organic compounds are produced by nature (Zhao et al. 2018). Synoptic studies (Kerminen et al. 2018; Nieminen et al. 2018) show the influence of natural condensation products on the GR level.

In this work, we have used data from high time-resolved particle number size distribution measurements to investigate any differences or similarities to GR and CS at four background stations located in different types of environments (urban, industrial, agricultural, and suburban) in the Czech Republic, Central Europe. The relations to the meteorological conditions and pollutants’ concentration were compared to find out which mechanisms are crucial at a particular kind of station.

## Methods

### Measurement sites

**Fig. 1:**
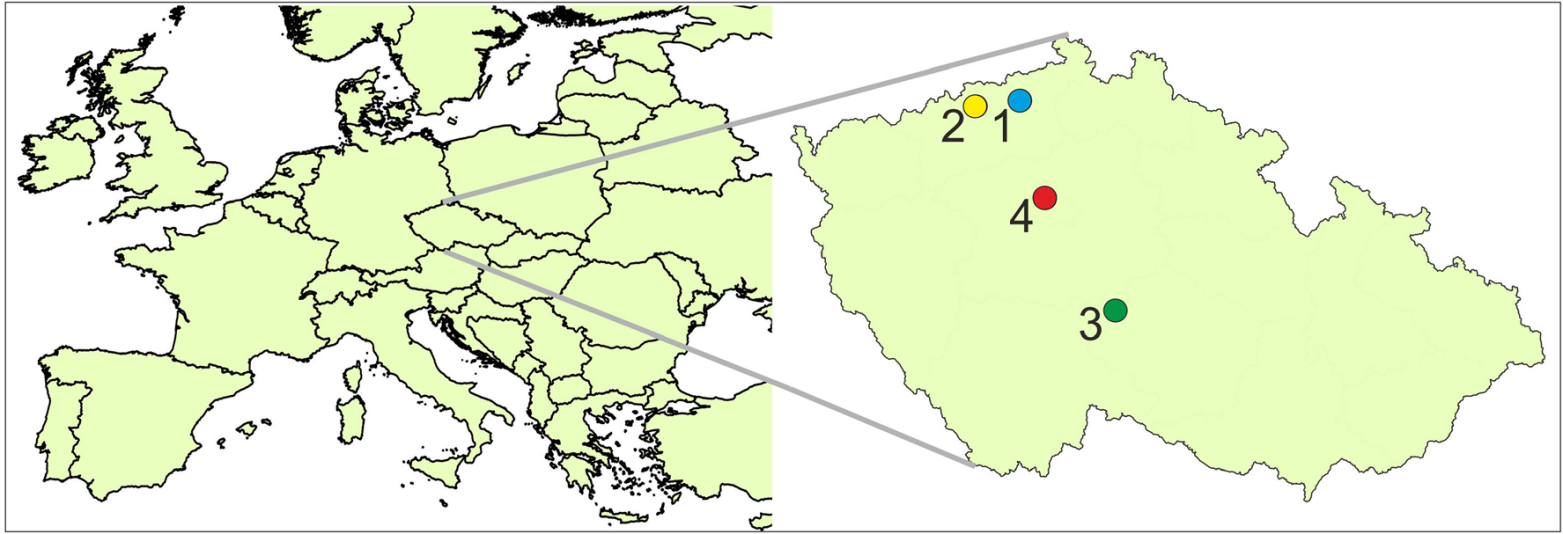
Location of the four background stations within the Czech Republic: (1) Ústí nad Labem-město, (2) Lom, (3) National Atmospheric Observatory Košetice, and (4) Prague-Suchdol

The measurements were carried at four background stations in the Czech Republic: Ústí nad Labem-město, Lom, National Atmospheric Observatory Košetice, and Prague-Suchdol (Fig. 1).

Ústí nad Labem-město and Lom stations are located in Ústí nad Labem region belonging to the zone called the “Black Triangle.” The Black Triangle covered the North-Bohemian Brown Coal Basin and parts of Silesia and Saxony. These localities were characterized by low air quality caused by large industrial sources of pollution. After the implementation of environmental laws at the beginning of the 1990s, the air quality started to improve. However, extensive opencast brown coal mining and the presence of large combustion sources and the petrochemical industry still adversely affect the air quality in the region as well as at both measurement sites (Hykyšová and Brejcha 2009).

Ústí nad Labem-město (Ústí n/L, 50° 39′ 39″ N, 14° 2′ 35″ E, 147 m a.s.l.) is an urban background station situated in a residential area 500 m from the town center. One hundred sixty meters north-east of the station is a road with a traffic volume of 21,000 cars per day (RSD 2020). A chemical company that specialized in synthetic resins, dyes, and chemical products (Ústí 2020) is located 1.3 km west of the station. The population of Ústí nad Labem is 93,000 inhabitants (CSO 2020).

Lom (50° 35′ 8″ N, 13° 40′ 24″ E, 265 m a.s.l.) is a rural background station. The opencast mine Bílina (1.8 km south-east from the measurement site) and a petrochemical complex (4 km south-west from the station) are located in the vicinity of the station. The complex is focused on a refinery and petrochemical product production (UNIPETROL 2020) and is the largest chemical production facility in the Czech Republic. The population of the nearest small town Lom (approximately 500 m north-west of the station) is 3,700 inhabitants, and the population of Litvínov at distance of 5 km north-west is 24,000 inhabitants (CSO 2020).

National Atmospheric Observatory Košetice (NAOK, 49° 34′ 24″ N, 15° 4′ 49″ E, 534 m a.s.l.) is a rural background station located in Bohemian-Moravian Highland. The agricultural landscape is composed of forest, fields, and meadows. NAOK is surrounded by several villages within a 3-km distance. Some of them have less than 30 inhabitants; in the largest village Košetice, there are 709 inhabitants (CSO 2020). A major highway D1 with a traffic volume of 40,000 cars per day (RSD 2020) is approximately 7 km NE of NAOK. A factory specialized in wood processing is located 7.5 km west of the sampling site.

Station Prague-Suchdol (Suchdol, 50° 7′ 35.507″ N 14° 23′ 4.700″ E, 277 m a.s.l.) is a suburban background station located in the campus of the Czech Academy of Sciences in a north-west residential part of Prague. Prague is the capital of the Czech Republic with 1.2 million inhabitants. The population of the residential quarter Suchdol is 5,800 inhabitants (CSO 2020). The traffic volume at the nearest road (200 m north of the site) is around 15,000 cars per day (Kubelová et al. 2015).

#### Emission inventories

Emission inventories for individual regions of the Czech Republic provided by the CHMI were used for local sources’ identification. This study deals with emissions of PM_10_ (Ústí n/L and Lom) or total suspended particles—TSP (NAOK and Suchdol), SO_2_, and NO_2_.

Since Ústí n/L and Lom are located in the same region, the emission inventory is the same for both stations. The main share of emissions was produced from coal mining and handling, public electricity and heat production, local heating, chemical industry, and traffic. NAOK emissions were dominantly composed of local heating, and traffic and agriculture activities. Emissions at Suchdol were characterized only by REZZO (Register of Emissions and Sources of Air Pollution) categories, without any possibility to distinguish among the individual sources. Despite this, the emissions of the agglomeration of Prague were mainly produced by local heating, traffic, and industry (CHMI 2019). Detailed graphs are in Supplementary Materials (Fig. S1).

### Instrumentation

Particle number size distributions (PNSD) were measured by scanning mobility particle sizers (SMPSs). The details of the instrumentation setting are listed in Table 1. The PNSD data were collected and proceeded according to the standards developed within the Aerosol, Clouds and Trace gases Research InfraStructure (ACTRIS) project. The measurements fulfilled all standard requirements and recommendations (Wiedensohler et al. 2012; WMO 2016). The instruments were regularly calibrated in the European Center for Aerosol Calibration (ECAC): the instruments at Ústí n/L and Lom twice per year, at NAOK, and Suchdol once every 2 years.

**Table 1:** Instrumental setup of PNSD measurement at the individual stations

Station	SMPS type	Condensation particle counter (CPC) type	Size range (nm)	Measurement interval (minute)
Ústí n/L	Custom-made*	TSI, 3772	10–800	5
Lom	Custom-made*	TSI, 3772	10–800	5
NAOK	Custom-made*	TSI, 3772	10–800	5
Suchdol	TSI 3034**	TSI, 3010	10–500	5

*Made by the Leibnitz Institute for Tropospheric Research

**Upgraded by the Leibnitz Institute for Tropospheric Research

### Data availability

At three stations (Ústí n/L, NAOK, and Suchdol), PNSD data were available from 2013 to 2017. At the Lom station, data were available only for 2017. The amount of missing data (Table 2) is connected to regular maintenances, calibrations in ECAC, and minor instrumentation failures. The evaluated period for all stations was set to the part of the year when the particle formation probability is higher, i.e., from March to October. Data from late autumn and winter were excluded because of the assumption that photochemical reactions would be suppressed as a result of low values of global radiation.

**Table 2:** PNDS data availability at the stations

Station	Evaluated period	Missing data (%)	NPF event days (%)
Ústí n/L	March–October 2013–2017	16	33
Lom	March–October 2017	13	40
NAOK	March–October 2013–2017	15	33
Suchdol	March–October 2013–2017	30	35

### Auxiliary data

Data from the National Air Quality Network (NAQM) operated by the Czech Hydrometeorological Institute (CHMI) were used to investigate air quality conditions at the stations. At each station, air quality data (sulfuric dioxide (SO_2_), nitrogen dioxide (NO_2_), and particulate matter (PM_10_)) were recorded by automatic analyzers. Also, the basic meteorological parameters (temperature (T), relative humidity (RH), wind speed (WS), wind direction (WD), and global radiation (GLB)) were measured directly at all the stations. The manufacturers and methods of the measurements mentioned above are shown in Table S1 in the Supplementary Materials (SM).

### Growth rate, condensation sink, and H_2_SO_4_ proxy calculation

Particle growth rate (GR) of freshly formed particles during NPF event days was calculated from the time evolution of the geometric mean diameter (GMD) values of particles smaller than 100 nm (Jeong et al. 2010):

1 $$ \mathrm{GR}=\frac{\Delta \mathrm{GMD}}{\Delta t} $$

where *∆t* is a time interval from the start to the end of growth and GMD was determined as (Hinds 1999):

2 $$ \mathrm{GMD}=\exp \frac{\sum \left(\ln {D}_{pi}\right)\times {N}_i\ }{\sum_i{N}_i} $$

where *D*_*pi*_ is the particle diameter of the size bin *i*, and *N*_*i*_ is the particle number concentration in the *i*th size bin.

Condensation sink (CS) determining the rate of molecule condensation (the loss of molecules) onto the pre-existing aerosol was computed by integrating the PNSD (Kulmala et al. 2004a):

3 $$ \mathrm{CS}=2\uppi D\int {D}_p{\upbeta}_M\ \left({D}_p\right)\ n\ \left({D}_p\right)\ d{D}_p=2\uppi D\sum {\upbeta}_M\ {D}_{pi}\ {N}_i $$

where *D* is the diffusion coefficient, *D*_*p*_ is the particle diameter, β_*M*_ is the transitional regime correction factor, *n* is the number concentration, *D*_*pi*_ is the particle diameter of size bin *i*, and *N*_*i*_ is the particle number concentration in the *i*th size bin.

For the calculation of diffusion coefficient *D*, we supposed sulfuric acid to be the primary condensing vapor in this region, as two stations (Ústí n/L and Lom) are located in the industrial region. Therefore, *D* was computed for sulfuric acid (${D}_{{\mathrm{H}}_2\mathrm{S}{\mathrm{O}}_4}$) as in (Pushpawela et al. 2018):

4 $$ {D}_{{\mathrm{H}}_2\mathrm{S}{\mathrm{O}}_4}=\left(5.0032\bullet {10}^{-6}\right)+\left(1.04\bullet {10}^{-8}T\right)+\left(1.64\bullet {10}^{-11}{T}^2\right)-\left(1.566\bullet {10}^{-14}{T}^3\right) $$

where *T* is the average temperature in Kelvin at the individual station from March to October during the studied period.

The transitional regime correction factor β_*M*_ was calculated according to the Fuchs-Sutugin equation (Fuchs and Sutugin 1971):

5 $$ {\upbeta}_M=\frac{\mathrm{Kn}+1}{1.33{\upalpha}^{-1}{\mathrm{Kn}}^2+1.33{\upalpha}^{-1}\mathrm{Kn}+0.38{\upalpha}^{-1}\mathrm{Kn}+1} $$

where Kn is Knudsen number equal to the ratio of the mean free path (ʎ = 66 nm) and the particle diameter *D*_*p*_ (Hinds 1999); the mass accommodation coefficient α was assumed to be 1, similarly to for example (Skrabalova et al. 2015).

Sulfuric acid (H_2_SO_4_) present in the atmosphere in gas phase is the key factor for NPF. H_2_SO_4_ in the gas phase is a product of SO_2_ oxidation by an OH radical (Zhang et al. 2011). In view of the fact that direct measurement of H_2_SO_4_ is not common in Czech Republic, the calculation of proxyH_2_SO_4_ concentrations by the equation (Petäjä et al. 2009) was used:

6 $$ \mathrm{proxy}{\mathrm{H}}_2{\mathrm{SO}}_4={k}_3\frac{\left[{SO}_2\right]\times GLB}{CS} $$

where *k*_3_ is the scaling factor (calculated according to Petäjä et al. 2009), [SO_2_] is the SO_2_ concentration, GLB is the global radiation, and CS is the condensation sink (calculated from Eq. 3).

### NPF event classification

Data were analyzed for NPF event occurrence according to the method by Dal Maso et al. (2005). Every day with available data was classified either as new particle formation event day (NPF), non-event day (NON), or undefined event day (UND) (Table 3). The day was classified as NPF if aerosol particle formation began in the nucleation mode (i.e., below 20 nm) and this new mode was recorded for more than 1 h with signs of growing. Only data recorded during NPF event days were used for further analysis.

**Table 3:** The number of classified events in individual months in the evaluated period

Station	Event/Month	III.	IV.	V.	VI.	VII.	VIII.	IX.	X.
Ústí n/L	NON	40	34	26	22	24	18	32	38
	NPF	*31*	*42*	*46*	*53*	*60*	*55*	*38*	*19*
	UND	70	57	57	45	40	42	65	72
	Total	141	133	129	120	124	115	135	129
Lom	NON	10	12	7	13	7	5	9	12
	NPF	*9*	*11*	*16*	*11*	*13*	*10*	*5*	*10*
	UND	12	5	7	6	6	4	8	6
	Total	31	28	30	30	26	19	22	28
NAOK	NON	61	34	25	28	40	32	51	60
	NPF	*48*	*49*	*48*	*52*	*51*	*49*	*30*	*19*
	UND	43	47	59	58	51	47	40	32
	Total	152	130	132	138	142	128	121	111
Suchdol	NON	19	19	8	16	6	9	12	24
	NPF	*36*	*40*	*49*	*47*	*30*	*50*	*34*	*17*
	UND	69	49	64	43	39	42	37	65
	Total	124	110	122	111	81	100	90	122

### Source location estimation

The potential location of sources influencing the growth rate values was computed in two ways, estimating both nearby and distant sources. Long-range transport was investigated through backward trajectories calculated by HYbrid Single-Particle Lagrangian Integrated Trajectory HYSPLIT_4 model (Draxler and Rolph 2013). Four-day backward trajectories were estimated every 6 h (00, 06, 12, 18 UTC), with Global Data Assimilation System (GDAS) meteorological data with 1° × 1° grid resolution used. The height of receptors was set to 500 m AGL. With the trajectories, the potential source contribution function (PSCF) was calculated by TrajStat plugin in MeteoInfoMap software (Wang et al. 2009). The PSCF estimates the probability of the source geographical location in a grid, with values close to 1 meaning the highest probability. The probability was calculated in 1° × 1° grid, the threshold value was set to median and 75th percentile of the GR, and a weighing function was applied (Zíková et al. 2016). The weighting factor determination is listed in Table S2 in the SM.

The local transport was estimated from the relation between wind speed, wind direction, and a selected variable, obtained by conditional probability function (CPF). The CPF polar plots, illustrating the probability of the occurrence of a concentration at given wind direction and wind speed, were calculated by the R package Openair (Carslaw and Ropkins 2012).

Air mass origin was analyzed from the backward trajectories clustered in the HYSPLIT model, based on the total spatial variance analysis.

## Results

### Basic overview

The atmospheric pollutant concentrations, condensation sink, and growth rate at each station were compared (Table 4). There were visible differences in pollution load between the stations related to the station’s location within the Czech Republic. At the Ústí n/L and Suchdol stations, close to towns, high concentrations of NO_2_ and PM_10_ and high values of CS were recorded. The highest levels of SO_2_ and GR were measured at the Lom station. The vicinity of the refinery complex may affect the mentioned values because of emission from the oil products’ processing. The rural background station NAOK reported the lowest amounts of all studied pollutants. Overview of the dependency of GR on selected meteorological elements, air pollutants, proxy H_2_SO_4_ concentration, and CS values is plotted in Fig. S2 and Fig. S3 in the Supplemental Materials. Constant GR values are visible over the whole measured range of most variables.

**Table 4:** Median, mean, and standard deviation (SD) of GR, air pollutants’ concentrations, values of CS, and proxy H_2_SO_4_ at Ústí n/L (*N* = 341), Lom (*N* = 86), NAOK (*N* = 346), and Suchdol (*N* = 302) in the period March–October 2013–2017

		GR	SO_2_	NO_2_	PM_10_	CS_H2SO4_	Proxy H_2_SO_4_
		(nm·h^−1^)	concentration (μg·m^−3^)			× 10^−3^ (s^−1^)	× 10^6^ (molecules·cm^−3^)
Ústí n/L	Median	3.85	4.15	20.40	20.79	13.44	0.65
	Mean	4.39	4.65	21.55	22.77	14.65	1.58
	SD	2.23	2.81	7.45	9.72	8.11	2.51
Lom	Median	4.20	5.77	7.83	22.04	9.40	2.36
	Mean	5.08	6.62	7.94	23.68	10.31	5.11
	SD	2.54	4.33	3.52	11.58	6.27	24.1
NAOK	Median	3.62	1.28	6.14	14.29	8.54	0.63
	Mean	4.13	1.60	6.55	15.77	9.14	0.87
	SD	2.24	1.15	2.53	6.97	4.51	0.83
Suchdol	Median	3.98	2.79	14.89	18.33	11.52	1.01
	Mean	4.47	3.36	17.82	20.46	12.99	1.48
	SD	2.30	2.00	9.66	9.78	7.67	1.62

### New particle formation and growth rate

**Fig. 2:**
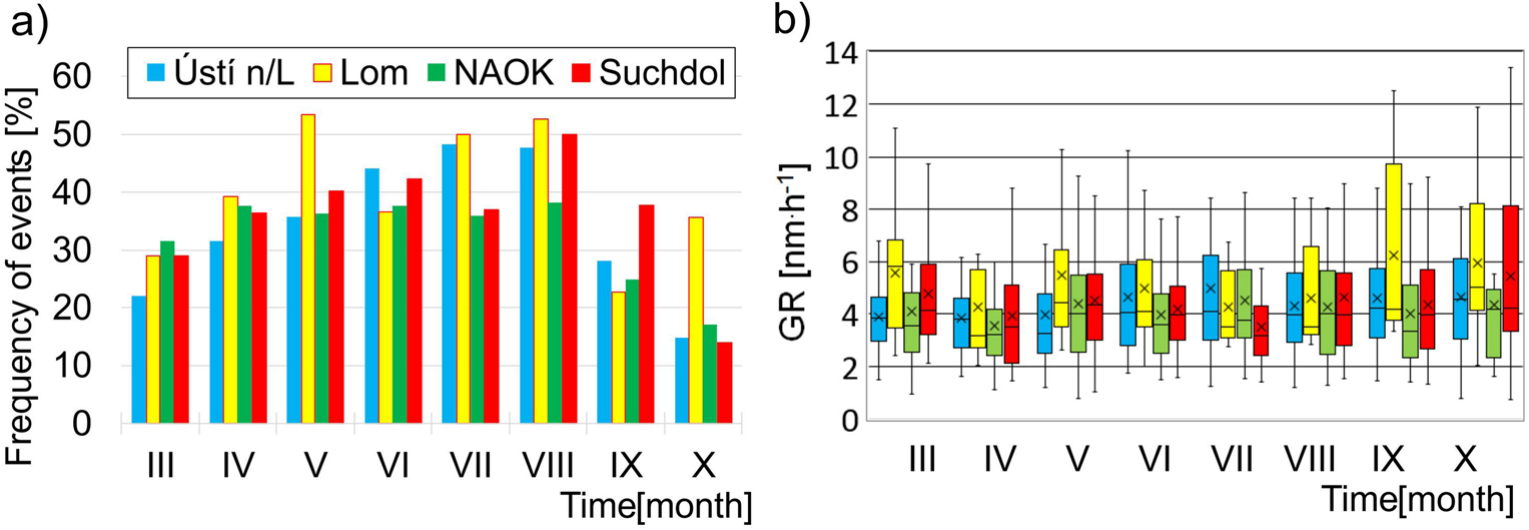
Monthly frequency of NPF event days (**a**) and monthly variability of GR (**b**) at Ústí n/L, Lom, NAO Košetice, and Suchdol in the period from March to October 2013–2017. In the box plot graph, the crosses show the average, horizontal lines indicate the median value, and the boxes denote the interquartile span

The annual cycle of NPF events did not follow the same pattern at all stations. NPF events frequency peaked in different months at individual stations—in July at Ústí n/L, in May at Lom, in June and August at NAOK, and in August at Suchdol. By contrast, the lowest NPF frequency was observed in October at almost all stations, except at Lom (Fig. 2a). As the Lom results are based on one season, however, it is possible that the same pattern as in other stations would be observed if data over a longer period were available.

The overall median GR was 3.9 ± 0.3 nm h^−1^ at all stations in the period under review. The minimal and maximal values of GR ranged from 0.77 to 17.34 nm h^−1^ at Ústí n/L, from 1.97 to 12.49 nm h^−1^ at Lom, from 0.77 to 15.70 nm h^−1^ at NAOK, and from 0.73 to 15.04 nm h^−1^ at Suchdol. Growth rates during NPF events fluctuated in individual months and stations as well. Whereas at Ústí n/L and NAOK the highest median GR was recorded in October, at the Suchdol station, the GR peaked in May and at the Lom station in March. On the contrary, the lowest median GRs were recorded at Lom and NAOK in April, in July at Suchdol, and in May at Ústí n/L (Fig. 2b). These results show relatively weak annual variability with no visible response to various seasons’ conditions.

### Condensation sink daily cycles

**Fig. 3:**
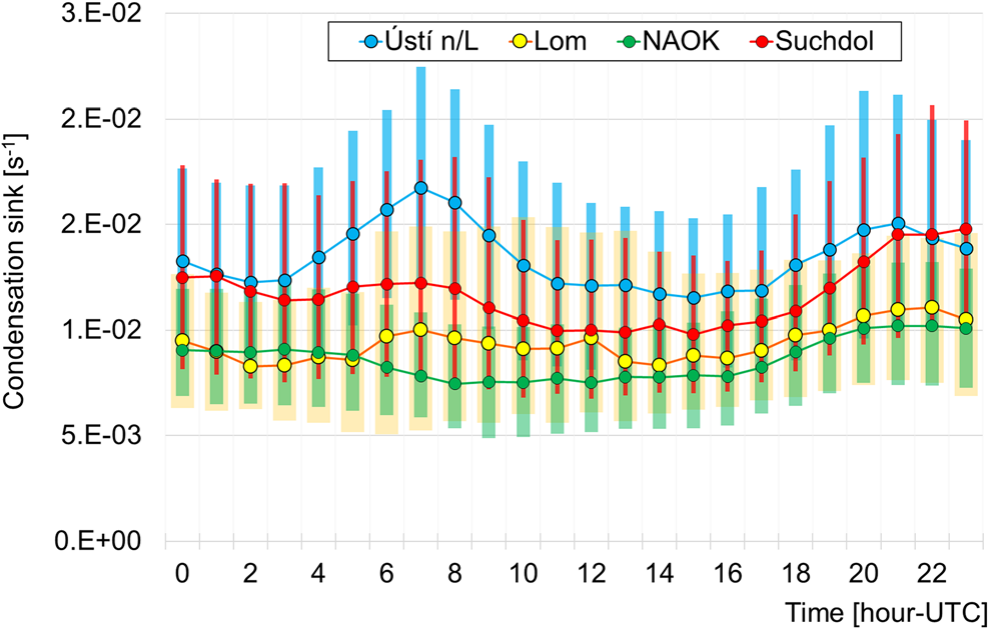
The median daily cycle of CS at stations the Ústí n/L, Lom, NAOK, Suchdol (denoted by markers). The top/bottom border of the boxes represents the 75th and 25th percentile, respectively

Each station was characterized by the daily cycle of CS, connected with pollutants’ loads. At the urban and suburban stations Ústí n/L and Suchdol stations, the connection with morning and evening traffic rush hours was quite pronounced (Fig. 3). In these two cases, increasing CS values after 3 AM peaking at 7 AM (CS–1.7·10^−2^ s^−1^ and 1.2·10^−2^ s^−1^) were observed. After reaching its maximum in the morning hours, CS decreased until the evening rush hour. The secondary maximum observed at all stations between 9 and 12 PM is a result of the decreasing boundary layer level.

At Lom, a different CS daily pattern was found. The decrease of values after morning peak continued only slowly, and a secondary maximum at noon was recorded (Fig. 3).

The response of CS to the atmospheric boundary layer (ABL) evolution was most visible at NAOK. CS dropped (9·10^−3^ s^−1^) after sunrise and kept its low values (7.5·10^−3^ s^−1^) until the evening hours (Fig. 3).

The evidence of pollutant influence on CS values is visible through normalized daily cycles of selected pollutants, namely by PM_10_ and NO_2_ concentration. The Pearson correlation coefficient between CS and PM_10_ was 0.56, 0.40, 0.50, and 0.67 at Ústí n/L, Lom, NAOK, and Suchdol, respectively. The relation between the PM_10_ and NO_2_ is expressed by the results of 0.77, 0.84, 0.80, and 0.83 for the same station order.

**Fig. 4:**
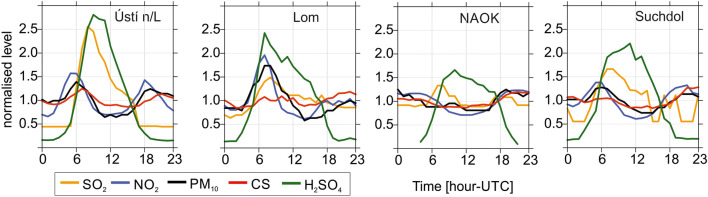
The normalized daily cycle of selected pollutants, CS and proxy H_2_SO_4_ at Ústí n/L, Lom, NAOK, and Suchdol

The diurnal variability of NO_2_ and PM_10_ and its connection to CS were very similar at Ústí n/L and Suchdol. Two peaks of NO_2_ and PM_10_ (amplitude 10–30 μg m^−3^) in the morning and evening hours were observed at these two stations, slightly more pronounced at Ústí n/L. On the contrary, at the Lom station, the response of CS to pollutant concentrations was different when compared to the other stations. Stable CS values did not follow well-developed morning peaks of pollutants (PM_10_–50 μg m^−3^, NO_2_–17 μg m^−3^, SO_2_–13 μg m^−3^). The secondary maximum of NO_2_ and PM_10_ in the evening was similar to the CS as at the rest of the stations. At NAOK, the highest pollutants’ concentrations were measured at night (apart from SO_2_), and the minimum around noon (PM_10_–15 μg m^−3^, NO_2_–5 μg m^−3^). SO_2_ daily variability behaved differently to NO_2_ and PM_10_ at all stations; a prevailing morning peak was recorded at Ústí n/L, Lom, and Suchdol, and morning and evening maxima occurred at NAOK. The SO_2_ daily cycle fluctuated at evening hours at Suchdol (Fig. 4).

The evolution of the H_2_SO_4_ concentrations began by a rapid increase from 3 AM reaching its maximum in the morning hours (Ústí n/L 2.71 × 10^6^, Lom 4.96 × 10^6^, NAOK 0.99 × 10^6^, and Suchdol 1.66 × 10^6^). Then H_2_SO_4_ concentrations started to decline steeply at Ústí n/L and Suchdol, gradually at Lom and NAOK until the evening hours. The boxplots showing the dependence of CS on selected meteorological elements, air pollutants, proxy H_2_SO_4_ concentration, and GR values are plotted in Fig. S4 and Fig. S5. CS was found to be constant over the whole range for the majority of the variables. Some increase in CS with increasing concentration is well pronounced for NO_2_ and PM_10_ at the Ústí n/L and Suchdol stations; such dependence was confirmed also by the Spearman correlation being over 0.5 (Fig. S6 in the SM).

Similarities in the daily cycles at Ústí n/L and Suchdol are probably caused by analogous sources of pollutants such as traffic and heating. The petrochemical factory and opencast possibly affect the pollutant concentrations at the Lom station. The weak daily evolution of the studied variables at NAOK is likely a result of the station’s location. Its position far from significant pollution sources enables the observation of the response of pollutants’ concentrations to ABL height development during the day, especially the dilution effect to atmospheric components.

### Growth rate patterns

Three categorized characteristics were used to describe the properties of GR at each station—the time of the beginning of the growth, length of the growth, and growth rate. The data were split into intervals (bins) representing a 2-h (or 2 nm h^−1^) period starting at zero. A particular bin contains the number of recorded occurrences of a specific range. The number of a score in the individual interval was divided by the total number of incidences for better comparison between the stations.

**Fig. 5:**
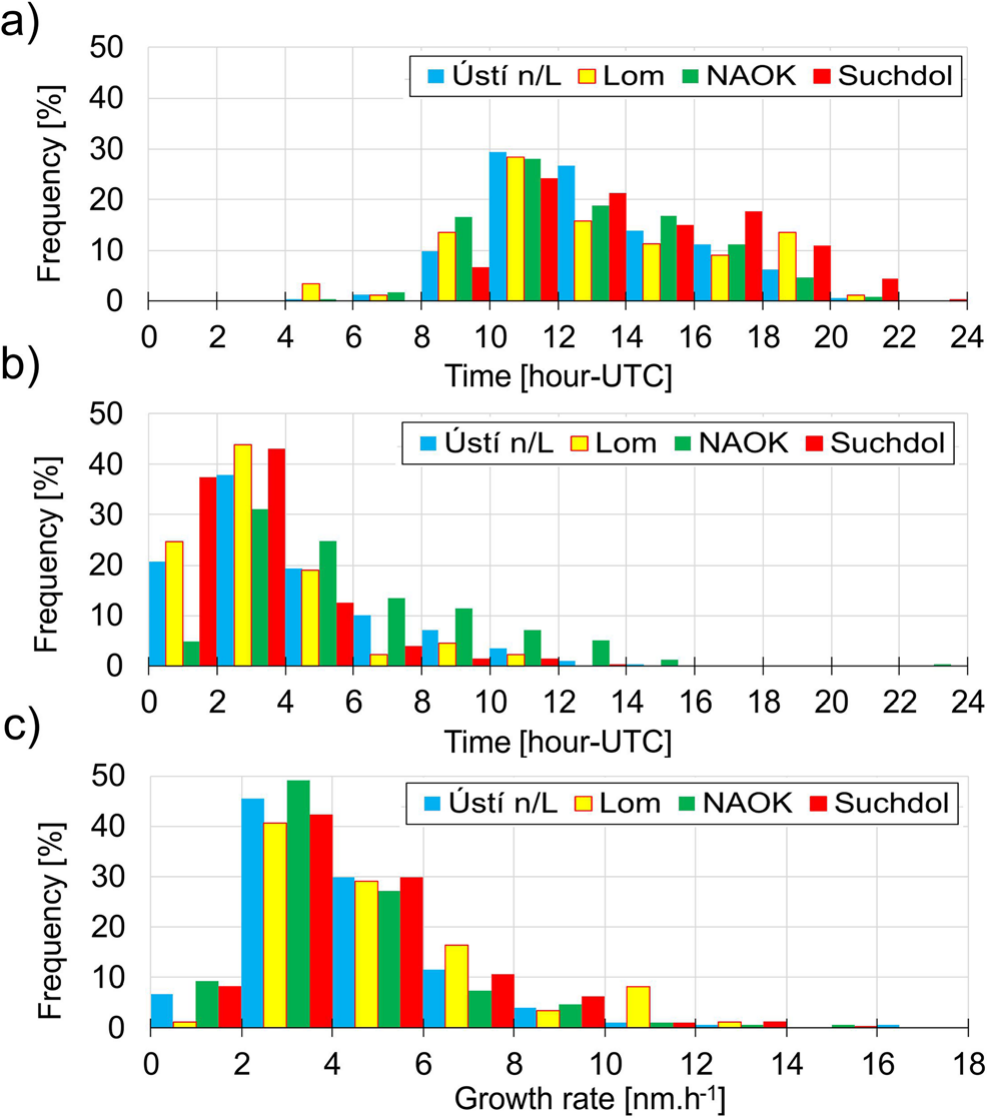
GR characteristics during NPF events at Ústí n/L, Lom, NAO Košetice, and Suchdol. **a** Frequency of the start time of the particle growth, **b** frequency of the length of the particle growth, **c** frequency of the growth rate

Some common patterns were found across the stations—the most frequent start time of the growth was from 10:00 to 12:00 UTC. However, individual behavior was observed as well. An increase of the frequency of the particle growth start time was recorded from 16:00 to 18:00 at Suchdol, and from 18:00 to 20:00 at Lom. At NAOK and Ústí n/L, the frequency of the GR start time continuously declined from its maximum between 10:00 and 12:00 (Fig. 5a).

Similarly to the time of the beginning, also the length of growth showed both similarities and differences at individual stations. The most typical length of growth was 2–4 h at all stations. The second highest frequency was recorded in length category 0–2 h, except for NAOK station with the 4–6 h being more frequent. Generally, at NAOK, longer lengths of growth were observed more often—growth frequently lasted up to 10 h, and NAOK was also the only station where growth took more than 12 h with a frequency of over 5% (Fig. 5b). The low value of CS may influence the GR observations. In a clean environment, such as at NAOK, existing particles do not inhibit the already-initiated growing process.In agreement with the median GR value close to 4 nm h^−1^ at all stations, the highest frequency of GR was in categories 2–4 and 4–6 nm h^−1^. At almost all stations, a gradual decrease in GR frequency in the direction toward upper classes was recorded. The GR frequency at Lom was different from other stations with some increase in GR over 10 nm h^−1^ (Fig. 5c).

### Local, regional, and long-ranged GR sources’ identification

#### Wind speed and direction dependence

CPF polar plots were used to represent the location of potential sources, mostly influencing the GR. Local and regional sources were estimated for events with a GR higher than the 75th percentile at the individual station, and the same analysis was also done for SO_2_, NO_2_, PM_10_, proxy H_2_SO_4_ concentrations, and CS values. For better orientation in the results, dashed red lines were added into polar plots, showing the spatial sector, where the probability of GR reaching 75th percentile is over 0.5.

At Ústí n/L, the strongest signal for the GR was identified from NNE wind directions with a wind speed of 4–6 m s^−1^. The probability of other variables in this direction is under 0.4. In contrast, a SO_2_ probability close to 1 was observed in the NE wind direction. In the NE direction, there is a crossroad with high traffic density and in the SE (GR second strongest signal) there is a power plant.

The strongest CPF signal at Lom was found from NE to SE directions. An opencast Bílina, a power plant, and two companies focused on glass production are at these locations. The results of the other studied pollutants do not correspond with the GR polar plot results. The probability of GR around 0.4 in the SW direction may be connected with the proximity of chemical factories which is confirmed by the SO_2_ and proxy H_2_SO_4_ results.

At NAOK, a probability close to 0.9 was recorded during NE winds with 8–10 m s^−1^ wind speed that is indicative of a regional source. One of the main Czech highways, D1, is located in the NE direction. The rest of the studied variables show probability over 0.3 associated with the NE direction, especially the PM_10_ and proxy H_2_SO_4_ results, which were between 0.6 and 0.8. However, a stronger signal was observed for SO_2_, CS, and proxy H_2_SO_4_ in the E direction.

**Fig. 6:**
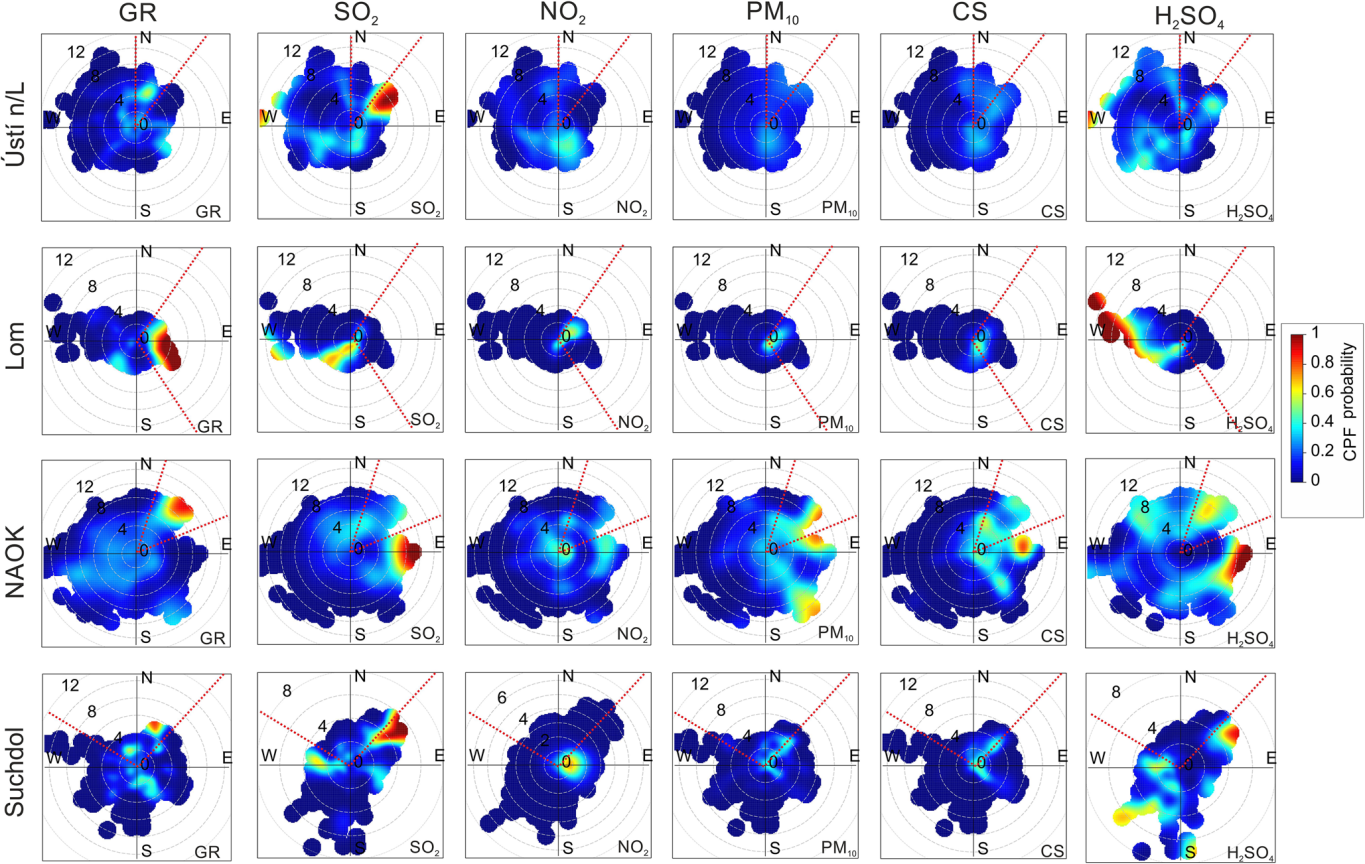
CPF polar plots of the probability of concentrations exceeding the 75th percentile of GR and hourly SO_2_, NO_2_, PM_10_ concentrations, CS, and proxy H_2_SO_4_ values connected with the wind direction and wind speed at Ústí n/L, Lom, NAO Košetice, and Suchdol. Dashed red lines indicate the spatial sector in the polar plots where the GR results exceeded 0.5 probability.

At the Suchdol station, a GR probability over 0.5 was recorded in some locations from NW to NE. These locations are sources like a road with high traffic density and local heating. The results of the other analyzed components not completely agree with the GR outcomes, but the SO_2_, PM_10_, CS, and proxy H_2_SO_4_ signals were over 0.4 in NE wind direction. The highest concentration of NO_2_ was associated with eastern winds with a low wind speed, which can be related to local sources. A proxy H_2_SO_4_ probability above 0.6 was recorded in the SW and S directions, which differs from the rest of the results (Fig. 6). Despite some similarities, it was not found that the GR at all stations is probably entirely affected by the same sources as the studied variables. Thus, either some additional pollutants would have to be considered or some of their combinations. Although the stations Ústí n/L, Lom, and Suchdol are close to emission sources, our results indicate that direct emissions of local sources do not affect GR substantially. Presumably, fresh emissions need to undergo chemical reactions to be effective for increasing GR. This assumption may be supported by the results from NAOK, where proxy H_2_SO_4_ showed a higher probability of occurrence (0.6) in the same direction as GR. NAOK is distant from direct pollution sources, unlike the other three stations.

#### Potential source location

The potential source contribution function (PSCF) analyses were done for a critical value set to the individual 75th percentile of GR at each station. Generally, similar regional sources and influence of long-range transport across the stations were found.

**Fig. 7:**
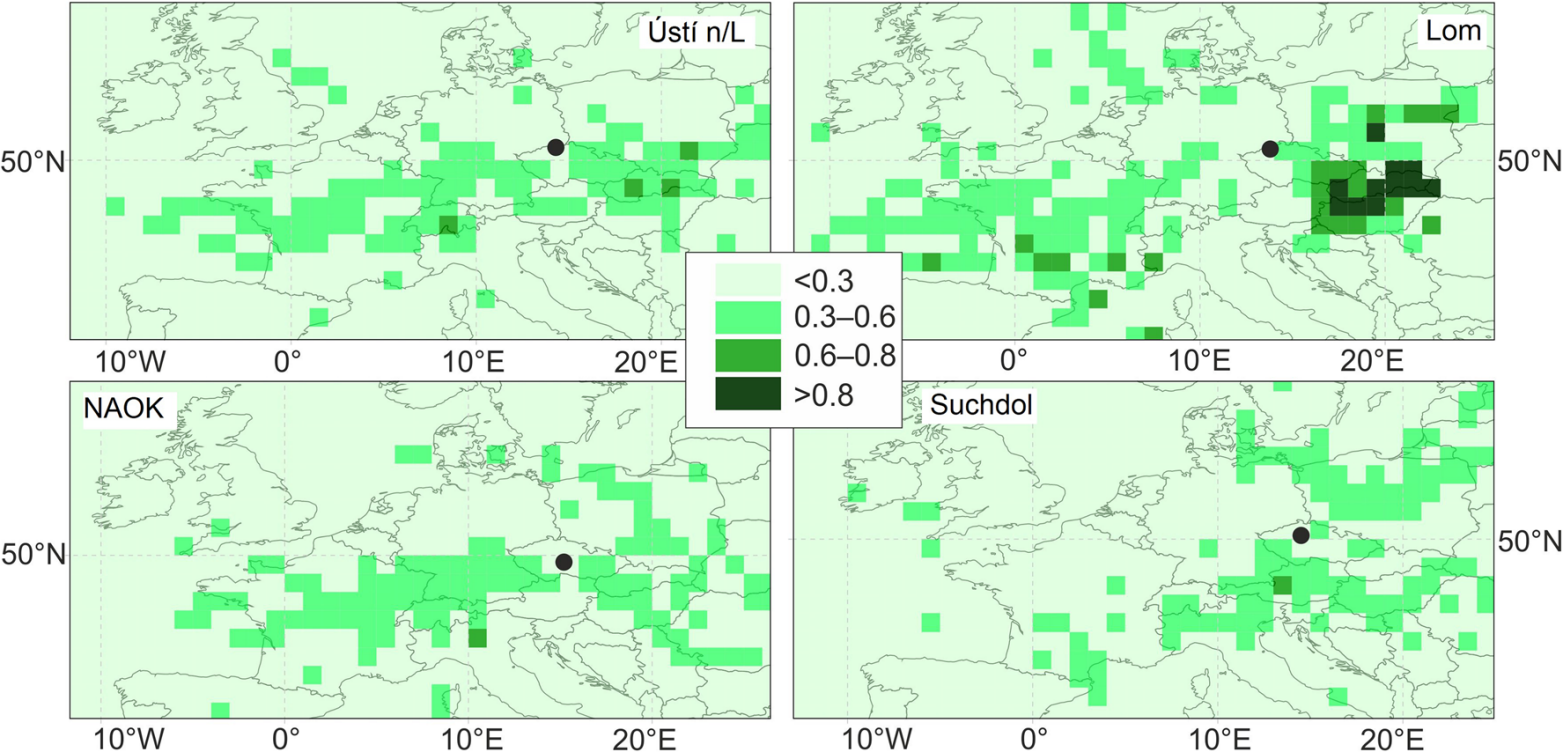
PSCF of the sources calculated for threshold set to 75th percentile of GR values. The black point indicates the station’s position—Ústí n/L, Lom, NAO Košetice, and Suchdol

Western sources located in line from Central France to Germany influence the GR values at Ústí n/L, Lom, and NAOK (probability 0.3–0.6). Only at Suchdol was no influence by western long-range transport seen (Fig. 7). The potential location of sources for all stations was identified in the north part of Italy, and also from the SE (probability 0.3–0.6). Suchdol and Lom were affected by regional/long-range transport from the NE through Poland.

The different probability of source contribution at Lom as compared to the rest of the stations was observed by eastern sources located in Poland, Austria, Hungary, and the Slovak Republic. Higher probabilities in the Lom results can be caused by a low number of trajectories, short data period, and also a small number of GR values, however.

Within the space of the Czech Republic, GR was influenced by SW, SE, and E sources. The east part of the Czech Republic is strongly influenced by heavy industry. Industrial complexes specialized in coal and steel processing are located close to the Czech-Polish border. Other industrial regions in the Czech Republic did not influence the GR values at the studied stations.

### Growth rates in different meteorological conditions and pollution loads

**Fig. 8:**
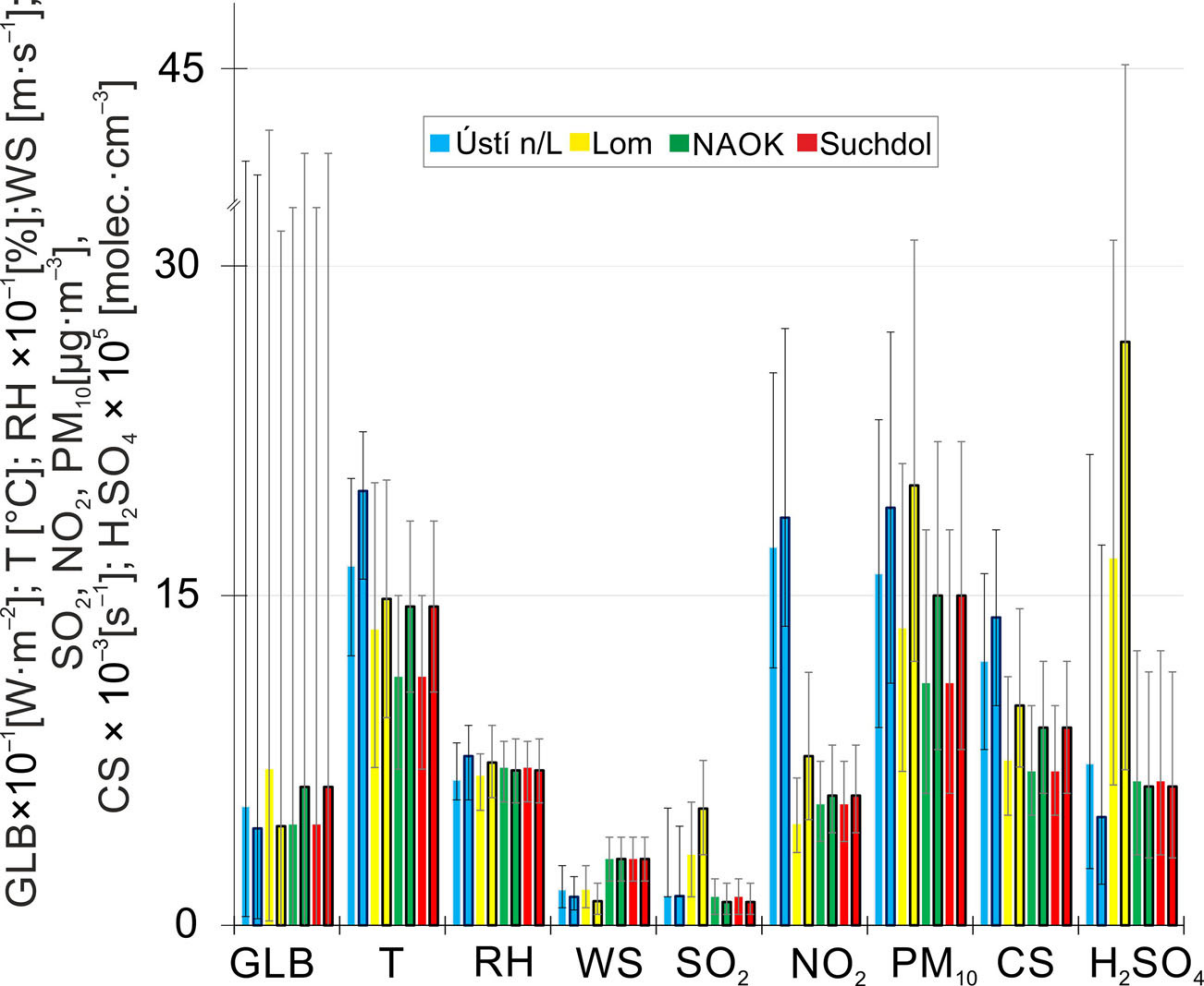
A statistical overview of meteorological parameters and pollutants’ concentrations during events when the measured GR was below the 25th (columns without outline borders), or above the 75th percentile values (columns with black outline borders), the top of the column is the median. The error bars show the 25th and 75th percentiles. GLB, global radiation; T, temperature; RH, relative humidity; WS, wind speed; SO_2_, sulfur dioxide; NO_2_, nitrogen dioxide; PM_10_, particulate matter; CS, condensation sink; H_2_SO_4_, H_2_SO_4_ proxy

The relation of the rate of growth and the atmospheric conditions was investigated based on the split dataset of all variables into two parts—events with GR below the 25th and above the 75th percentile (Fig. 8). The differences in the meteorological parameters or pollutants’ concentrations in the two datasets were tested by the Mann-Whitney *U* non-parametric test. No statistically significant difference was found for the meteorological variables (global radiation, wind speed and wind direction, and temperature) except global radiation at Ústí n/L. These results indicate that the observed meteorological conditions probably do not strongly influence the GR values. The meteorological conditions were similar when GR was below the 25th and above the 75th percentiles. Two cases representing differences between the two groups in pollutants’ concentrations were recorded for SO_2_ at Lom and for NO_2_ at Suchdol station. These results indicate that the observed meteorological conditions do not strongly influence the GR values. Although the pollutants’ concentrations were usually higher in the GR > 75th percentile dataset (apart from SO_2_ at NAOK and Suchdol and proxy H_2_SO_4_ at Ústí n/L, NAOK, and Suchdol), there was probably an additional (not observed) component (e.g., VOC, OH radical, condensable vapor concentration, aerosol chemical composition (O’Dowd et al. 2002; Petäjä et al. 2009; Zhang et al. 2011; Kulmala et al. 2017)) in the atmosphere that controls the growth rate.

The results from the Spearman correlation coefficient applied on the two datasets did not show any similarities across the stations. The strength between variables differs from station to station, and no general conclusion is evident (Fig. S6 in SM).

**Fig. 9:**
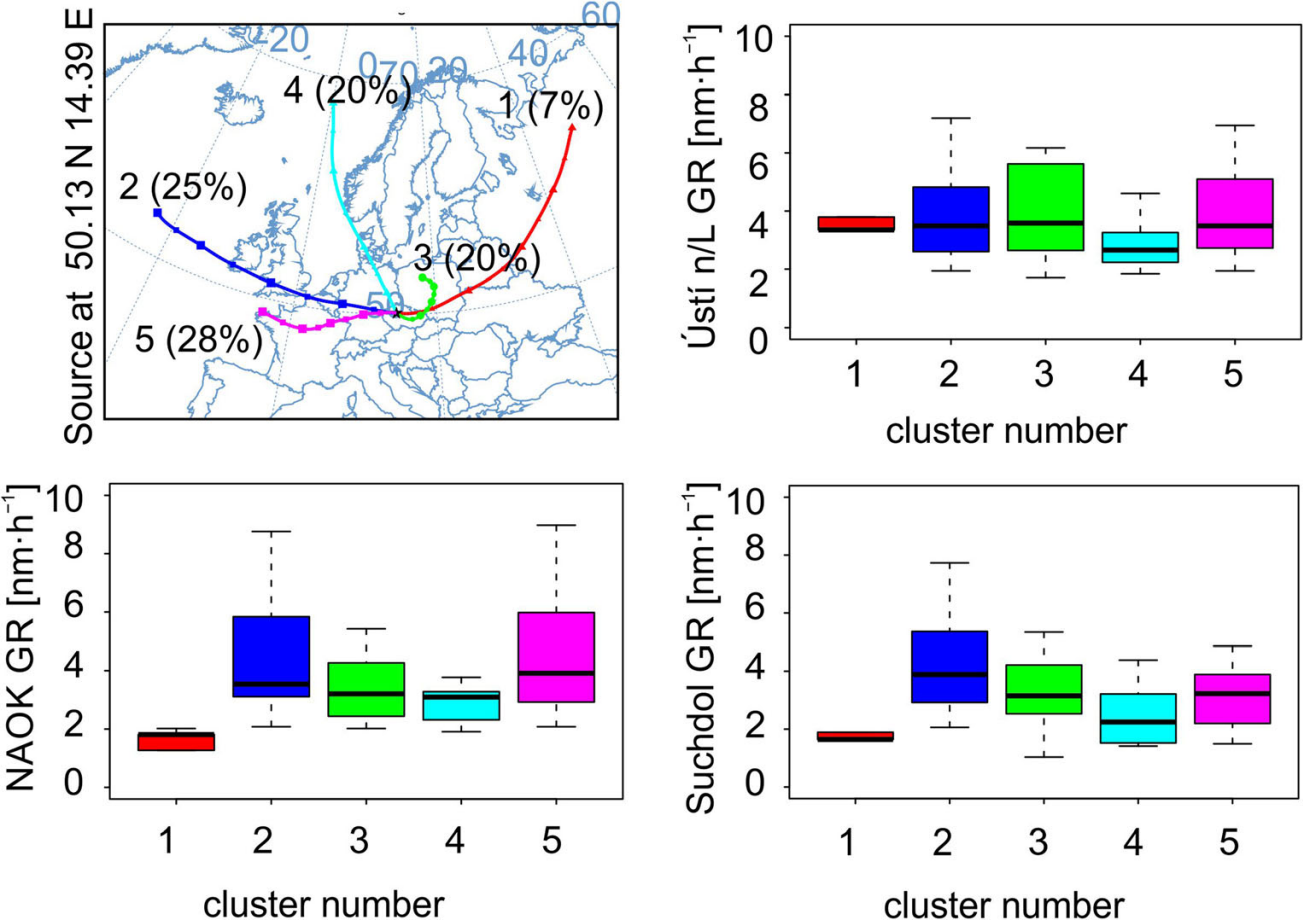
Statistical cluster analysis of air mass backward trajectories showing the dependency of GR on different air mass origin at Ústí n/L, NAOK, and Suchdol. One receptor site, Suchdol, was chosen, as the distance between stations is smaller than 100 km, the spatial resolution of the model. Cluster number 1—fresh continental; 2—fresh marine; 3—aged continental; 4—fresh marine; and 5—continental air mass. The boxes are colored the same as clusters, the black horizontal line is the median, the borders of the boxes show the 25th and 75th percentiles, and the error bars indicate the minimum and maximum values

As a proxy for aerosol composition (that was not available for most of the stations), the air mass origin was considered and the GR was compared in various air masses. As the HYSPLIT model resolution was smaller than the geographical distance of the stations, only one station was selected as a receptor site—the Suchdol station, which represents the middle of the Czech Republic—for the clustering. For the air mass analyses, only days with the NPF occurrence at all stations were included in the calculation (53 days). The Lom station was excluded for this part because of the lack of data (only 6 days). The total amount of 198 backward trajectories were clustered to 5 clusters. Cluster numbers 1, 3, and 5 were of continental origin (number 3 is aged air mass), and clusters 2 and 4 are of fresh marine origin (Fig. 9). Throughout all stations, the results of GR in different air masses were similar. Cluster number 1 included only several days, so the statistics are not very good. The highest GR median values were observed in clusters 5 (continental), and 2 (fresh marine), apart from Ústí n/L, where in cluster 3 (aged continental) the median GR was slightly higher. The lowest median GR occurred in cluster 4 (fresh marine). Thus, no clear signal in the connection between the GR and continental or maritime air masses, with different chemical composition, that would be applicable for all three stations was found.

## Summary and discussion

The goal of the study was to investigate differences and/or similarities influencing the formation of aerosol particles and their consequent growth in different types of the background environments. This evaluation is based on GR and CS characteristic during NPF events.

Although NPF events’ occurrence was considered to be more frequent in clean areas (Kulmala et al. 2017; Kerminen et al. 2018), these findings do not apply to the GR and CS values. The observation in megacities proved frequent NPF events, which is not in line with theory. Another physical and chemical mechanisms influence this process in a mixture of urban pollution (Kulmala et al. 2017). Similar results were found in our data; NPF occurred also at the most polluted station Lom. According to review by Kerminen et al. (2018), less polluted areas are typical for lower GR compared to highly polluted locations; the contrary is true for CS. An abundance of condensable materials, manifested by higher CS values, probably causes increased GR in polluted areas (Bousiotis et al. 2019; Chu et al. 2019).

At our background stations, the differences in the CS daily evolution reflecting the type of environment were found. The response to anthropogenic activities (morning and evening rush hours) was recorded at Ústí n/L and Suchdol; unique CS evolution, probably connected to close mining activities, occurred at Lom. At the cleanest location, i.e., NAOK, the linkage between pollution dilution and ABL daily development was visible.

The calculated GR values referring to a median value around 4 nm h^−1^ correspond to the results of similar stations type reported in recent studies (Bousiotis et al. 2019; Kerminen et al. 2018; Nieminen et al. 2018). A unique GR length time lasting more than 10 h with frequency over 10% was recorded at NAOK.

Potential local and regional sources influencing GR were associated with directions where pollution sources are situated. The links with the sources of other studied pollutants were confirmed. The proxy H_2_SO_4_ at NAOK suggests that very close direct emissions may need to undergo a chemical reaction with other atmospheric components to act on GR. Only at this station, thanks to the absence of direct pollution sources, was it possible to identify the same source site of the studied variables (in particular for PM_10_, CS, and proxy H_2_SO_4_) as for GR. Alternatively, some other atmospheric species probably affect GR. Regional and long-range transport seem to influence GR similarly.

The investigated similarities and differences at the stations reflect the influence of the type of surrounding environment, local topography, and the character of the emissions. Although all stations are background, we can observe the same patterns that are usually more pronounced in the more polluted areas. Our results show that among background stations, the found differences can be related to specific pollutions and these findings can be helpful for other comparisons and further studies of NPF events.

## Supplementary Information

ESM 1 (DOCX 1899 kb)

## Data Availability

The datasets generated during and/or analyzed during the current study are available from the corresponding author on reasonable request.

## References

[CR1] Bousiotis D, Osto MD, Beddows DCS et al (2019) Analysis of new particle formation (NPF) events at nearby rural, urban background and urban roadside sites. Atmos Chem Phys 19(8):5679–5694

[CR2] Carslaw DC, Ropkins K (2012). openair -- an R package for air quality data analysis. Environ Model Softw.

[CR3] CHMI (2019). Air pollution in the Czech Republic in 2018.

[CR4] Chu B, Matti Kerminen V, Bianchi F (2019). Atmospheric new particle formation in China. Atmos Chem Phys.

[CR5] CSO (2020) Population of municipalities - 1 January 2019. https://www.czso.cz/csu/czso/population-of-municipalities-1-january-2019. Accessed 14 Aug 2020

[CR6] Dada L, Paasonen P, Nieminen T, Buenrostro Mazon S, Kontkanen J, Peräkylä O, Lehtipalo K, Hussein T, Petäjä T, Kerminen VM, Bäck J, Kulmala M (2017). Long-term analysis of clear-sky new particle formation events and nonevents in Hyytiälä. Atmos Chem Phys.

[CR7] Dal Maso M, Kulmala M, Riipinen I (2005). Formation and growth of fresh atmospheric aerosols: eight years of aerosol size distribution data from SMEAR II, Hyytiälä, Finland. Boreal Environ Res.

[CR8] Draxler RR, Rolph GD (2013). HYSPLIT (HYbrid Single-Particle Lagrangian Integrated Trajectory).

[CR9] Fuchs NA, Sutugin AG (1971). Topics in current aerosol research (part 2), high dispersed aerosols, Pard 2.

[CR10] Hamed A, Joutsensaari J, Mikkonen S, Sogacheva L, Dal Maso M, Kulmala M, Cavalli F, Fuzzi S, Facchini MC, Decesari S, Mircea M, Lehtinen KEJ, Laaksonen A (2007). Nucleation and growth of new particles in Po Valley, Italy. Atmos Chem Phys.

[CR11] Hinds W (1999). Aerosol technology : properties, behavior, and measurement of airborne particles.

[CR12] Hykyšová S, Brejcha J (2009). Monitoring of PM10 air pollution in small settlements close to opencast mines in the North-Bohemian Brown Coal Basin. WIT Trans Ecol Environ.

[CR13] Jeong CH, Evans GJ, McGuire ML (2010). Particle formation and growth at five rural and urban sites. Atmos Chem Phys.

[CR14] Kerminen V, Chen X, Vakkari V et al (2018) Atmospheric new particle formation and growth: review of field observations. Environ Res Lett 13(10):103003

[CR15] Kuang C, Chen M, Zhao J, Smith J, McMurry PH, Wang J (2012). Size and time-resolved growth rate measurements of 1 to 5 nm freshly formed atmospheric nuclei. Atmos Chem Phys.

[CR16] Kubelová L, Vodička P, Schwarz J, Cusack M, Makeš O, Ondráček J, Ždímal V (2015). A study of summer and winter highly time-resolved submicron aerosol composition measured at a suburban site in Prague. Atmos Environ.

[CR17] Kulmala M, Petäjä T, Mönkkönen P, Koponen IK, Dal Maso M, Aalto PP, Lehtinen KEJ, Kerminen VM (2004). On the growth of nucleation mode particles: source rates of condensable vapor in polluted and clean environments. Atmos Chem Phys Discuss.

[CR18] Kulmala M, Vehkamaki H, Petaja T (2004). Formation and growth rates of ultrafine atmospheric particles: a review of observations. J Aerosol Sci.

[CR19] Kulmala M, Kerminen V-M, Petäjä T, Ding AJ, Wang L (2017). Atmospheric gas-to-particle conversion : why NPF events are observed in megacities ?. Faraday Discuss.

[CR20] Ling Y, Wang Y, Duan J, Xie X, Liu Y, Peng Y, Qiao L, Cheng T, Lou S, Wang H, Li X, Xing X (2019). Long-term aerosol size distributions and the potential role of volatile organic compounds (VOCs) in new particle formation events in Shanghai. Atmos Environ.

[CR21] Nie W, Ding A, Wang T, Kerminen VM, George C, Xue L, Wang W, Zhang Q, Petäjä T, Qi X, Gao X, Wang X, Yang X, Fu C, Kulmala M (2014). Polluted dust promotes new particle formation and growth. Sci Rep.

[CR22] Nieminen T, Kerminen V-M, Petäjä T (2018). Global analysis of continental boundary layer new particle formation based on long-term measurements. Atmospheric Chem Phys.

[CR23] O’Dowd CD, Hämeri K, Mäkelä J et al (2002) Coastal new particle formation: environmental conditions and aerosol physicochemical characteristics during nucleation bursts. J Geophys Res 107(D19). 10.1029/2000JD000206

[CR24] Petäjä T, Mauldin RL, Kosciuch E et al (2009) Sulfuric acid and OH concentrations in a boreal forest site. Atmospheric Chem Phys 9(19):7435–7448. 10.5194/acp-9-7435-2009

[CR25] Pikridas M, Sciare J, Freutel F, Crumeyrolle S, von der Weiden-Reinmüller SL, Borbon A, Schwarzenboeck A, Merkel M, Crippa M, Kostenidou E, Psichoudaki M, Hildebrandt L, Engelhart GJ, Petäjä T, Prévôt ASH, Drewnick F, Baltensperger U, Wiedensohler A, Kulmala M, Beekmann M, Pandis SN (2015). In situ formation and spatial variability of particle number concentration in a European megacity. Atmos Chem Phys.

[CR26] Pöschl U (2005). Atmospheric aerosols: Composition, transformation, climate and health effects. Angew Chemie - Int Ed.

[CR27] Pushpawela B, Jayaratne R, Morawska L (2018). Temporal distribution and other characteristics of new particle formation events in an urban environment. Environ Pollut.

[CR28] RSD (2020) Celostátní sčítání dopravy 2016. http://scitani2016.rsd.cz/pages/informations/default.aspx. Accessed 10 Aug 2020

[CR29] Skrabalova L, Zikova N, Zdimal V (2015). Shrinkage of newly formed particles in an urban environment. Aerosol Air Qual Res.

[CR30] Stocker TF, Qin D, Plattner GK et al (2013) IPCC, 2013: climate change 2013: the physical science basis. In: Contribution of Working Group I to the Fifth Assessment Report of the Intergovernmental Panel on Climate Change. Cambridge University Press, Cambridge and New York, pp 1535

[CR31] UNIPETROL (2020) Chempark Záluží. https://www.unipetrolrpa.cz/en/ServicesandChempark/ChemparkZaluzi/Pages/default.aspx. Accessed 10 Aug 2020

[CR32] Ústí nad L (2020) Economy. https://www.usti-nad-labem.cz/en/city/introduction/economy.html. Accessed 14 Aug 2020

[CR33] Wang YQ, Zhang XY, Draxler RR (2009). TrajStat: GIS-based software that uses various trajectory statistical analysis methods to identify potential sources from long-term air pollution measurement data. Environ Model Softw.

[CR34] Wiedensohler A, Birmili W, Nowak A, Sonntag A, Weinhold K, Merkel M, Wehner B, Tuch T, Pfeifer S, Fiebig M, Fjäraa AM, Asmi E, Sellegri K, Depuy R, Venzac H, Villani P, Laj P, Aalto P, Ogren JA, Swietlicki E, Williams P, Roldin P, Quincey P, Hüglin C, Fierz-Schmidhauser R, Gysel M, Weingartner E, Riccobono F, Santos S, Grüning C, Faloon K, Beddows D, Harrison R, Monahan C, Jennings SG, O'Dowd CD, Marinoni A, Horn HG, Keck L, Jiang J, Scheckman J, McMurry PH, Deng Z, Zhao CS, Moerman M, Henzing B, de Leeuw G, Löschau G, Bastian S (2012). Mobility particle size spectrometers: harmonization of technical standards and data structure to facilitate high quality long-term observations of atmospheric particle number size distributions. Atmos Meas Tech.

[CR35] WMO (2016) WMO/GAW aerosol measurement procedures, guidelines and recommendations, 2nd edn. World Meteorological Organization GAW Rep. 227, pp 93. https://library.wmo.int/opac/doc_num.php?explnum_id=3073

[CR36] Yli-Juuti T, Nieminen T, Hirsikko A, Aalto PP, Asmi E, Hõrrak U, Manninen HE, Patokoski J, Dal Maso M, Petäjä T, Rinne J, Kulmala M, Riipinen I (2011). Growth rates of nucleation mode particles in Hyytiälä during 2003-2009: variation with particle size, season, data analysis method and ambient conditions. Atmos Chem Phys.

[CR37] Zhang R, Khalizov A, Wang L (2011). Nucleation and growth of nanoparticles in the atmosphere. Chem Rev.

[CR38] Zhang J, Chen Z, Lu Y, Gui H, Liu J, Wang J, Yu T, Cheng Y (2016). Observations of new particle formation, subsequent growth and shrinkage during summertime in Beijing. Aerosol Air Qual Res.

[CR39] Zhao C, Li Y, Zhang F, Sun Y, Wang P (2018). Growth rates of fine aerosol particles at a site near Beijing in June 2013. Adv Atmos Sci.

[CR40] Zíková N, Wang Y, Yang F, Li X, Tian M, Hopke PK (2016). On the source contribution to Beijing PM2.5 concentrations. Atmos Environ.

