# Supplementary material for: Atmospheric aerosol growth rates at different background station types
Source: Environ Sci Pollut Res Int. 2020 Nov 12;28(11):13352–64. doi: 10.1007/s11356-020-11424-5 (PMC7943522; doi:10.1007/s11356-020-11424-5)
Supplement: Supplementary file 1 — (DOCX 1899 kb) [file 11356_2020_11424_MOESM1_ESM.docx]

# Supplementary materials:

# Atmospheric aerosol growth rates at different background station types

Adéla HOLUBOVÁ ŠMEJKALOVÁ^a,b^, Naděžda ZÍKOVÁ^b,c^, Vladimír ŽDÍMAL^c^ , Helena PLACHÁ^a^, Miroslav BITTER^a^

^a^ Czech Hydrometeorological Institute, Na Šabatce 2050/17, 143 06 Prague 4-Komořany,
 Czech Republic

^b^ Institute for Environmental Studies, Faculty of Science, Charles University, Benátská 2,
 128 01 Prague 2, Czech Republic

^c^ Institute of Chemical Process Fundamentals, CAS, Rozvojová 135, 165 02 Prague 6,
 Czech Republic

Table S1. Instrumentation manufacturers and measurement methods.

| **Station.**  **Variable…....** | | **Ústí n/L** | **Lom** | **NAOK** | **Suchdol** |
| --- | --- | --- | --- | --- | --- |
| **SO_2_** | instrument | Thermo Environmental Instruments, M43 | Teledyne Advanced Pollution Instrumentation T100* | Teledyne Advanced Pollution Instrumentation T100* | Thermo Environmental Instruments, M43 |
|  | *method* | *UV-fluorescence* | *UV-fluorescence* | *UV-fluorescence* | *UV-fluorescence* |
| **NO_2_** | instrument | Teledyne Advanced Pollution Instrumentation, T200* | Teledyne Advanced Pollution Instrumentation, T200* | Teledyne Advanced Pollution Instrumentation, T200* | Thermo Environmental Instruments, M42 |
|  | *method* | *chemi-luminescence* | *chemi-luminescence* | *chemi-luminescence* | *chemi-luminescence* |
| **PM_10_** | instrument | Thermo ESM Andersen, FH 62 I-R/ Environnement SA, MP101M** | Thermo ESM Andersen, FH 62 I-R/ Environnement SA, MP101M** | Thermo ESM Andersen, FH 62 I-R/ Environnement SA, MP101M** | Thermo ESM Andersen, FH 62 I-R/ Environnement SA, MP101M** |
|  | *method* | *radiometry - beta ray absorption* | *radiometry - beta ray absorption* | *radiometry - beta ray absorption* | *radiometry - beta ray absorption* |
| **T, RH** | instrument | Thies HTT Compact* | Thies HTT Compact* | Vaisala HMP 155 | Commet System, NH 421 |
|  | *method* | *resistance method* | *resistance method* | *resistive platinum sensors* | *resistance method* |
| **WS** | instrument | WindSonic-Gill Instruments Ltd. | WindSonic-Gill Instruments Ltd. | Vaisala WAA 251 | WindSonic-Gill Instruments Ltd. |
|  | *method* | *ultrasonic anemometer* | *ultrasonic anemometer* | *optoelectronic anemometer* | *ultrasonic anemometer* |
| **WD** | instrument | WindSonic-Gill Instruments Ltd. | WindSonic-Gill Instruments Ltd. | Vaisala WAV 151 | WindSonic-Gill Instruments Ltd. |
|  | *method* | *ultrasonic anemometer* | *ultrasonic anemometer* | *optoelectronic wind vane* | *ultrasonic anemometer* |
| **GLB** | instrument | TM, SG 420 | TM, SG 420 | Kipp-zonen CMP-11 | TM, SG 420 |
|  | *method* | *temperature difference method* | *temperature difference method* | *temperature difference method* | *temperature difference method* |

* until 2015, the same instrument as in Suchdol was used, ** in operation from 2016

Table S2. The criterion for Weighing function used for PCSF analysis.

| Station | Criterion of weight | | |
| --- | --- | --- | --- |
|  | 3×$\bar{n}$ | 1.5×$\bar{n}$ | 0.75×$\bar{n}$ |
| Ústí n/L | 96 | 48 | 24 |
| Lom | 24 | 21 | 6 |
| NAOK | 96 | 48 | 24 |
| Suchdol | 84 | 42 | 21 |

| $W_{i,j}$= | $\left\{ \begin{aligned} 1.00 \\ 0.72 \\ 0.42 \\ 0.17 \end{aligned} \right.$ | $\left. \begin{aligned} 3\bar{n} < 3n_{i,j} \\ 3\bar{n} > n_{i,j}\geq1.5\bar{n} \\ 1.5\bar{n} > n_{i,j}\geq0.75\bar{n} \\ 0.75\bar{n} > n_{i,j} \end{aligned} \right\}$ |
| --- | --- | --- |

Notes: the meaning of used symbols is $\bar{n}$ = the average number of trajectory endpoints in each cell; $n_{i,j}$=the number of trajectory endpoints that fell into a geographically defined cell


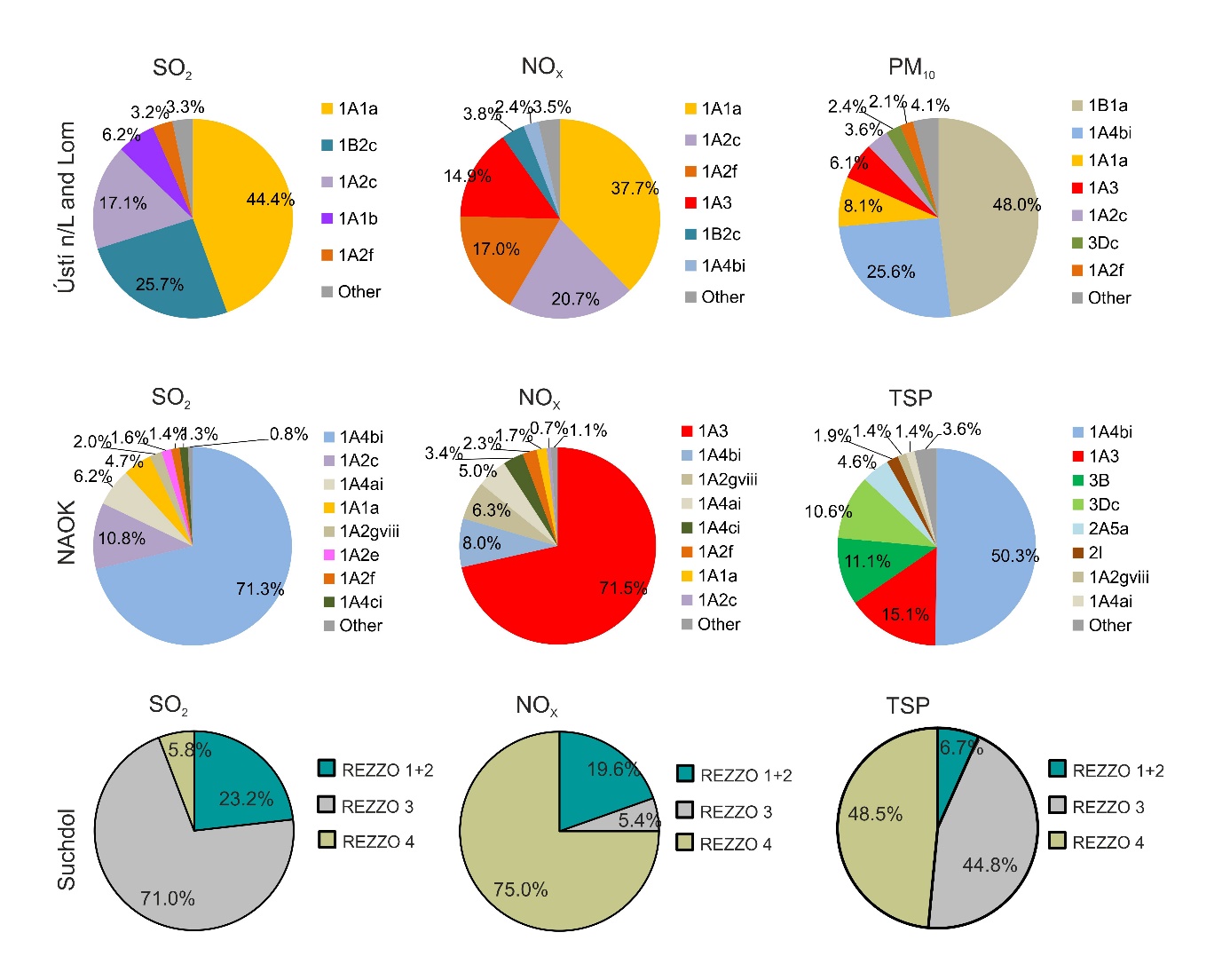


**Fig. S1** Emissions of selected pollutants listed according to Nomenclature for reporting (NFR) and Register of Emissions and Sources of Air Pollution (REZZO) at Ústí n/L, Lom, NAO Košetice, and Suchdol in 2016. The The legends are listed in Tables S1 and S2. Note: Ústí n/L and Lom are located in the same region, the emission inventory is the same for both stations.

Table S3. Description of used codes of Nomenclature for reporting (NFR) of emission.

| **NFR code** | **Name of category** |
| --- | --- |
| 1A1a | Public electricity and heat production |
| 1A1b | Petroleum refining |
| 1A2c | Stationary combustion in manufacturing industries and construction: Chemicals |
| 1A2e | Stationary combustion in manufacturing industries and construction: Food processing, beverages and tobacco |
| 1A2f | Stationary combustion in manufacturing industries and construction: Non-metallic minerals |
| 1A2gviii | Stationary combustion in manufacturing industries and construction: Other |
| 1A3 | Transport |
| 1A4ai | Commercial/institutional: Stationary |
| 1A4bi | Residential: Stationary |
| 1A4ci | Agriculture/Forestry/Fishing: Stationary |
| 1B1a | Fugitive emission from solid fuels: Coal mining and handling |
| 1B2c | Venting and flaring (oil, gas, combined oil and gas) |
| 2A5a | Quarrying and mining of minerals other than coal |
| 2I | Wood processing |
| 3B | Livestock |
| 3Dc | Farm-level agricultural operations including storage, handling  and transport of agricultural products |

Table S4. The classification of air pollution sources according to the method of emission monitoring REZZO categories.

| **Category** | **Description** |
| --- | --- |
| REZZO 1+2 | Stationary combustion plants for combustion of fuels with a total heat consumption 0.3 MW and higher, waste incinerators, other sources (technological combustion processes, industrial production etc.) |
| REZZO 3 | Stationary combustion plants for combustion of fuels with a total nominal heat consumption and lower 0.3 MW, non-specified technological processes (domestic solvent use etc., building and agricultural activities) |
| REZZO 4 | Road, railway, water and air transport of persons and freight, for road transport also tire and brake wear, road abrasion and evaporation from fuel systems of vehicles using petrol, operation of off-road vehicles and machines used in maintenance of green spaces in parks and forests etc. |


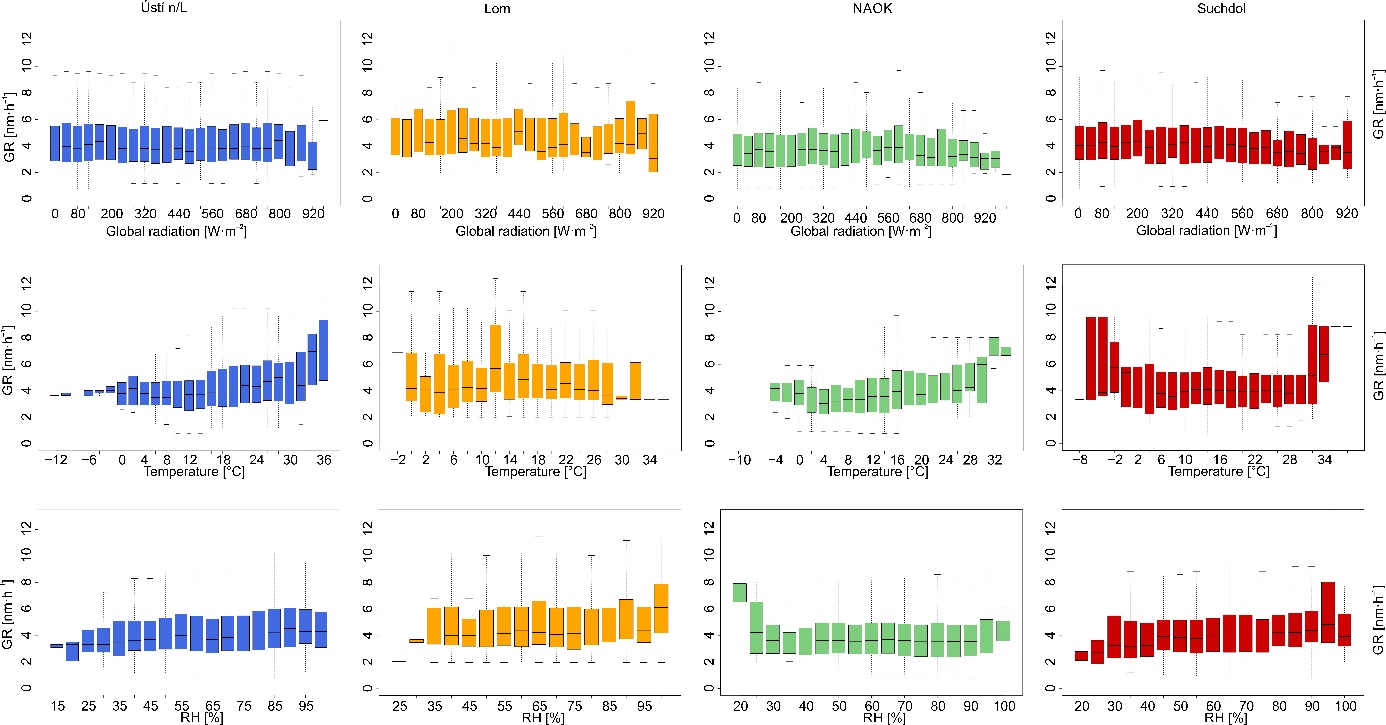


**Fig. S2** Boxplots of global radiation, temperature, relative humidity, during different levels of GR. The black horizontal line is the median, the borders of the boxes show 25^th^ and 75^th^ percentiles, the error bars indicate the minimum and maximum values.


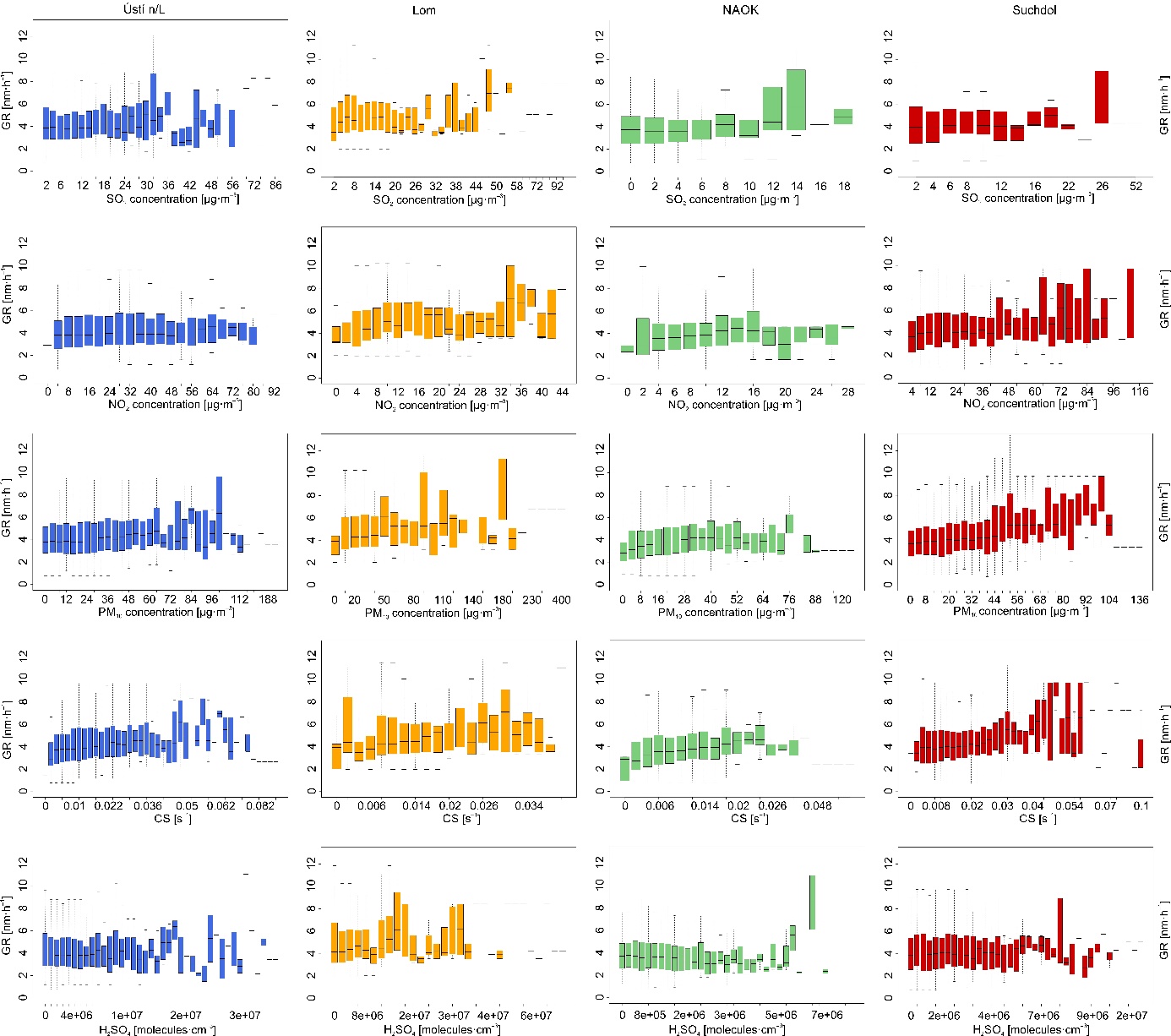


**Fig. S3** Boxplots of SO_2_ concentration, NO_2_ concentration, PM_10_ concentration, CS, and H_2_SO_4_ proxy during different levels of GR. The black horizontal line is the median, the borders of the boxes show the 25^th^ and 75^th^ percentiles, the error bars indicate the minimum and maximum values.


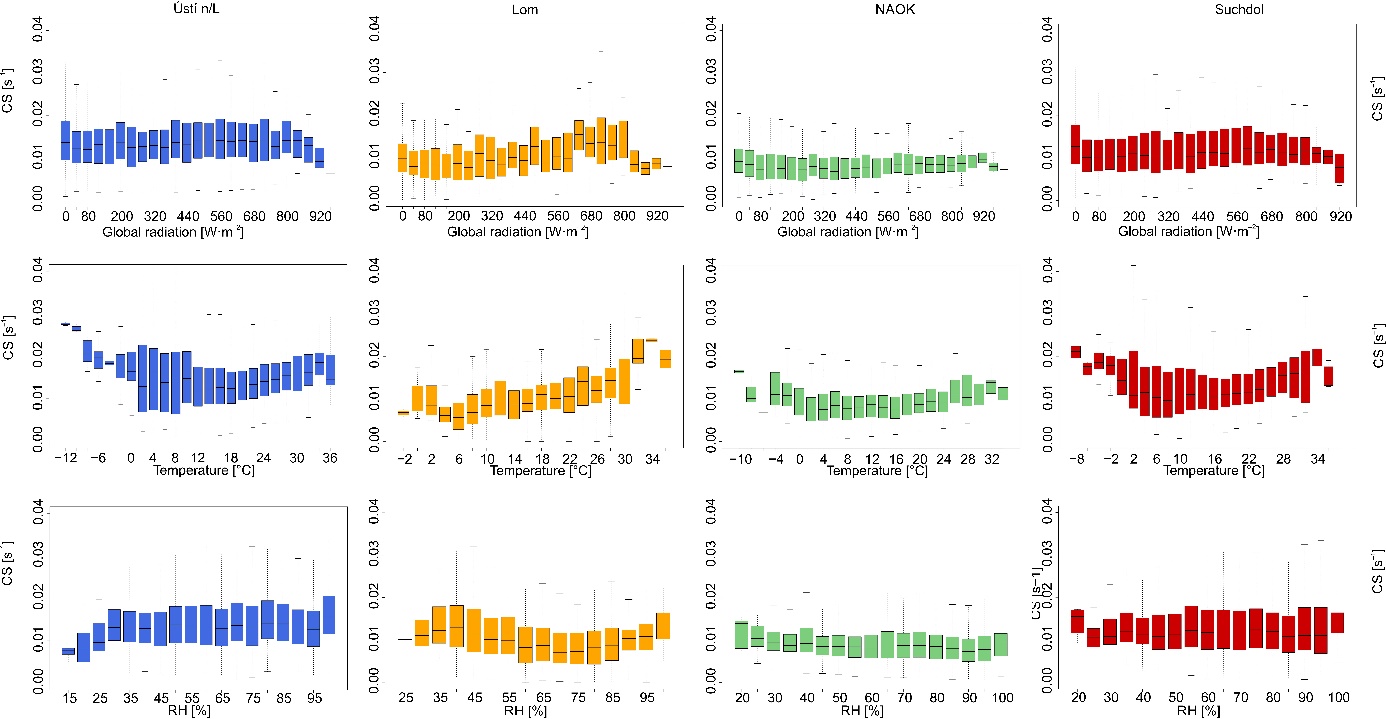


**Fig. S4** Boxplots of global radiation, temperature, relative humidity during different levels of CS. The black horizontal line is the median, the borders of the boxes show the 25^th^ and 75^th^ percentiles, the error bars indicate the minimum and maximum values.


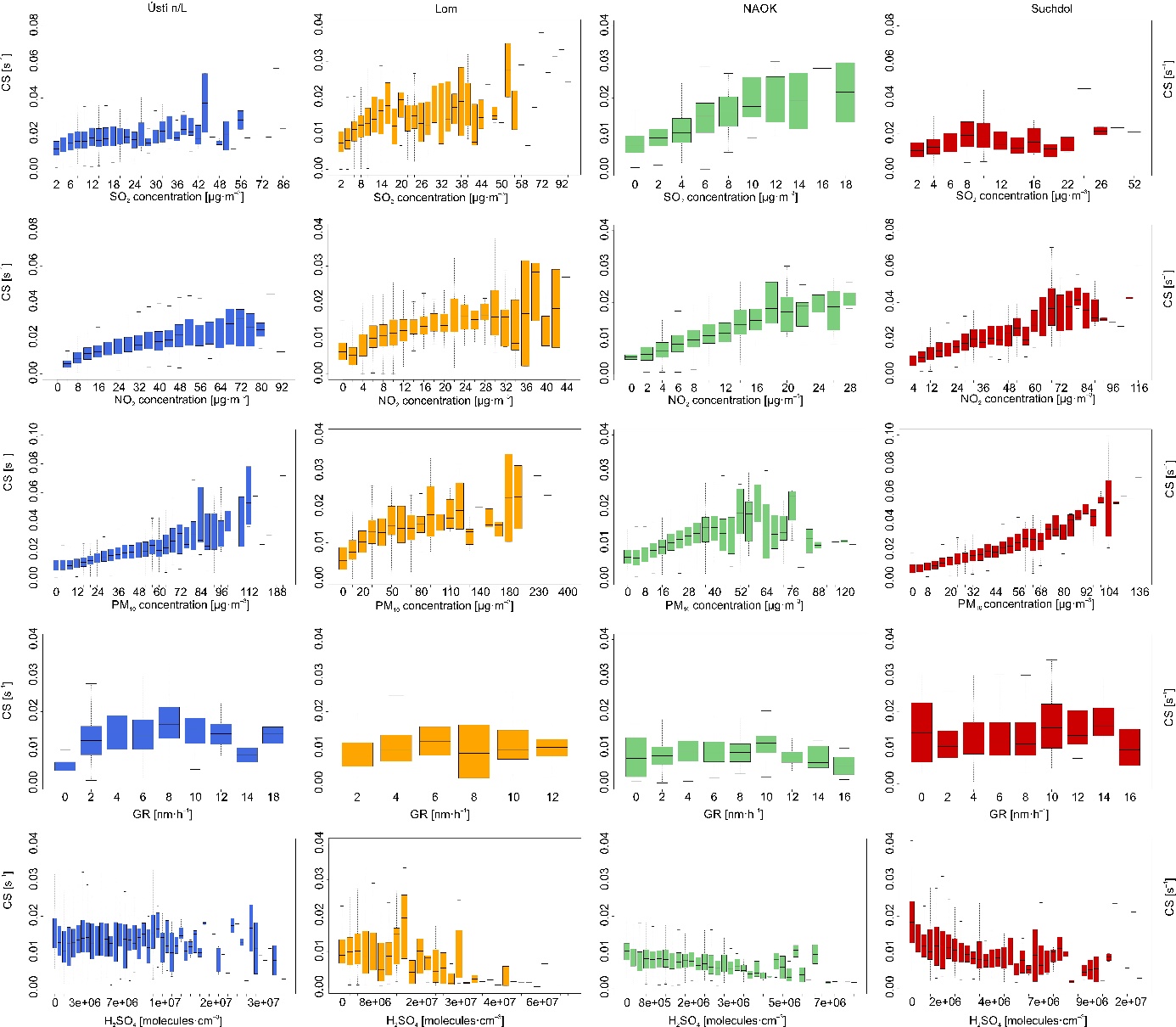


**Fig. S5** Boxplots of SO_2_ concentration, NO_2_ concentration, PM_10_ concentration, GR, and H_2_SO_4_ proxy during different levels of CS. The black horizontal line is the median, the borders of the boxes show the 25^th^ and 75^th^ percentiles, the error bars indicate the minimum and maximum values. Note: The CS scale for SO_2_ NO_2_ PM_10_ concentration is different at Ústí n/L and Suchdol station.


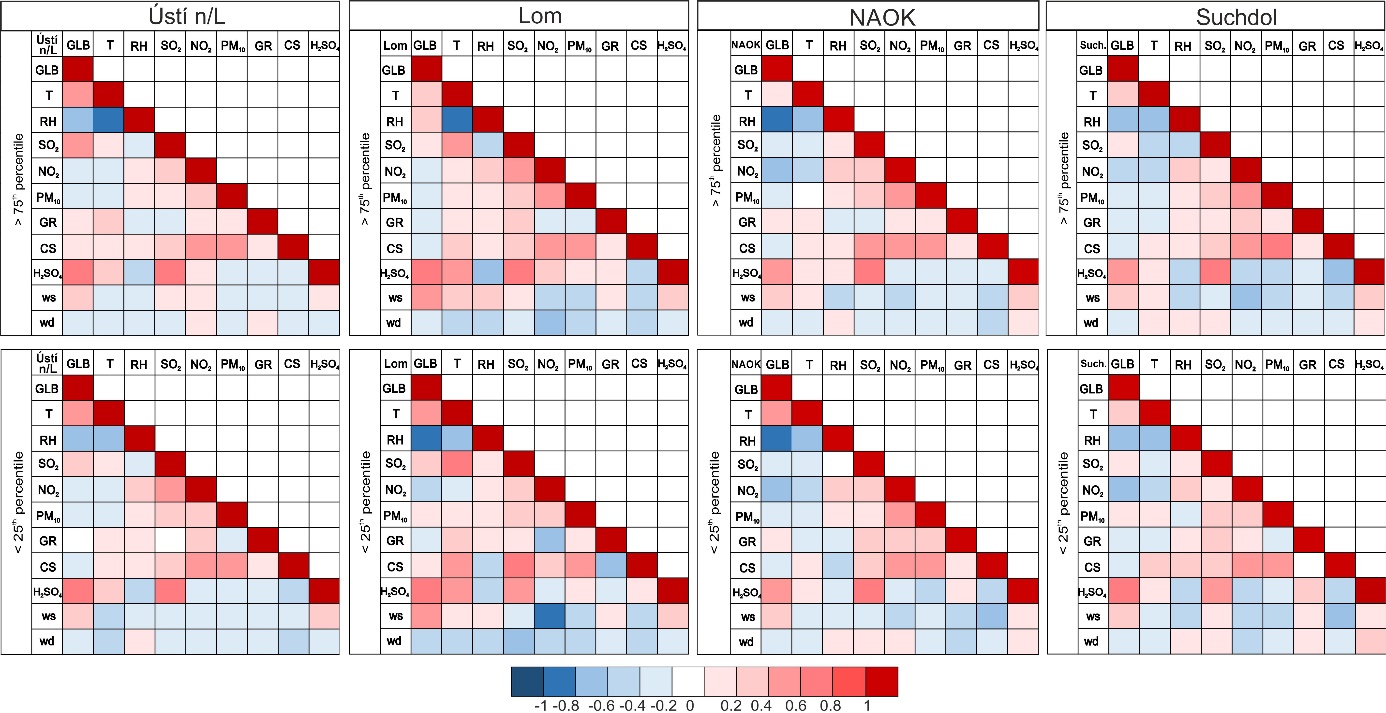


**Fig. S6** Spearman correlation diagrams for meteorological parameters and pollutants concentrations during events when the measured GR were below the 25^th^ and above 75^th^ percentile values at Ústí n/L, Lom, NAO Košetice and Suchdol.
